# Causes and Effects of Oocyte Retrieval Difficulties: A Retrospective Study of 10,624 Cycles

**DOI:** 10.3389/fendo.2021.564344

**Published:** 2022-01-03

**Authors:** Yang Wang, Meixiang Zhang, Hao Shi, Shiqi Yi, Qian Li, Yingchun Su, Yihong Guo, Linli Hu, Jing Sun, Ying-pu Sun

**Affiliations:** ^1^ Center for Reproductive Medicine, The First Affiliated Hospital of Zhengzhou University, Zhengzhou, China; ^2^ Henan Key Laboratory of Reproduction and Genetics, The First Affiliated Hospital of Zhengzhou University, Zhengzhou, China

**Keywords:** assisted reproduction, clinical pregnancy rate, cumulative pregnancy rate (CPR), cumulative live birth rate, oocyte retrieval difficulty

## Abstract

Oocyte retrieval is a routine procedure during the application of assisted reproduction technology. However, technical difficulties experienced during oocyte retrieval and the subsequent unsatisfactory number of oocytes obtained are rarely reported. The current study included 10,624 oocyte retrieval cycles from April 2015 to June 2018, and patients were followed up until February 2019. Patients were divided into two groups depending on whether the oocyte number obtained reached the >14-mm follicle number on the day of hCG administration. In the oocyte retrieval not satisfactory (ORNS) group, there were 1,294 cycles, and in the oocyte retrieval satisfactory (ORS) group, there were 9,330 cycles. ORNS patients were older, had a longer duration of infertility, had higher follicle-stimulating hormone, and were more likely to have endometriosis. The ORS group had a higher rate of the use of a follicular phase long-acting gonadotropin-releasing hormone (GnRH) agonist long ovarian stimulation protocol and a lower rate of the use of a luteal phase short-acting GnRH agonist long protocol. The ORNS group had fewer total number of days of FSH stimulation. On human chorionic gonadotropin day, the ORNS group had higher luteinizing hormone (LH), lower estradiol, and lower progesterone levels. After oocyte retrieval, the oocyte quality and fresh cycle transplantation rate were higher in the ORNS group. An unsatisfactory oocyte retrieval number did not influence the clinical pregnancy rate, miscarriage rate, or live birth rate during the fresh cycles. The cumulative pregnancy rate and the live birth rate were lower in the ORNS group. In conclusion, with a similar number of matured follicles, ORNS was more likely to occur in ovarian dysfunction patients. The follicular phase long-acting GnRH agonist long protocol had lower oocyte retrieval difficulty during IVF/ICSI. ORNS does not affect embryo quality or the fresh cycle pregnancy rate, but it significantly reduces the cumulative pregnancy rate and the live birth rate.

## Introduction

Transvaginal oocyte retrieval is a regular procedure in each *in vitro* fertilization (IVF)/intracytoplasmic sperm injection (ICSI) cycle. The aim is to obtain matured oocyte–cumulus complexes after controlled ovarian stimulation. Sometimes, however, despite the operator trying to obtain oocytes from each >14-mm follicle, an unsatisfactory number of oocytes is retrieved.

Neither the reasons for oocyte retrieval difficulty nor the effects that arise from it have been thoroughly investigated. Follicular flushing has been used in attempts to negate unsatisfactory oocyte retrieval. In patients in whom follicular aspiration was difficult, oocytes could reportedly be obtained by repeated follicular flushing. In a survey of Australian assisted reproduction technology (ART) units, >50% of operators used follicular flushing in addition to direct aspiration during oocyte retrieval (1). Follicular flushing increased the number of oocytes retrieved (2), but in randomized controlled IVF/ICSI trials, direct aspiration and follicular flushing were associated with similar outcomes, including the numbers of oocytes retrieved, fertilization rates, embryo quality, and pregnancy rates in both normal and poor ovarian responders (3–10). Thus, follicular flushing has become a safe and efficient way to increase the number of oocytes obtained.

Oocyte retrieval difficulty usually manifested as the number of oocytes acquired after four to six follicular flushes remains unsatisfactory. Not all of these patients are poor ovarian responders. Some patients even have more than 10 follicles according to our observation. The reasons for this phenomenon are unclear, and few studies have focused on this population. Only endometriosis has been definitively identified as a cause of oocyte retrieval difficulty (11), and this causative association may be related to insufficient cumulus expansion, where cumulus expansion-relative genes were decreased in endometriosis patients (12). Our investigation showed that patients with unsatisfactory oocyte retrieval numbers (i.e., the final oocyte number obtained was less than the >14-mm follicle number) were accounted for 12.18% of all IVF/ICSI cycles. The reasons for unsatisfactory oocyte retrieval numbers and its influence on ART outcome remain to be determined.

## Materials and Methods

### Study Design and Population

Women undergoing IVF or ICSI at the First Affiliated Hospital of Zhengzhou University were enrolled in the study. The study was conducted from February 2015 to June 2018 and was approved by the Independent Ethics Committee of Zhengzhou University. Patients with a body mass index >15 and <36, between the ages of 22 and 45 years, with an indication for IVF or ICSI, and presenting with at least one follicle >14 mm in both ovaries combined were considered eligible for inclusion. The exclusion criteria were mini cycles or natural stimulation cycles, no oocyte unoccupied cycles, or preimplantation genetic diagnosis cycles. A total of 10,624 oocyte retrieval cycles were included in the study.

Final oocyte maturation was conventionally induced by 6,500 IU recombinant human chorionic gonadotropin (hCG) and 2,000 IU urinary hCG. In patients at high risk of ovarian hyperstimulation syndrome, 6,500 IU recombinant hCG or 3,000–5,000 IU urinary hCG was used. Oocyte pickup was scheduled 34–37 h thereafter. The numbers and sizes of follicles were assessed and recorded just before beginning the retrieval process. The main aim was to procure follicles with diameters >14 mm. Oocyte retrieval was performed together with oocyte pickup under a microscope by two embryologists. Follicles >14 mm were aspirated or flushed.

**Figure 1:**
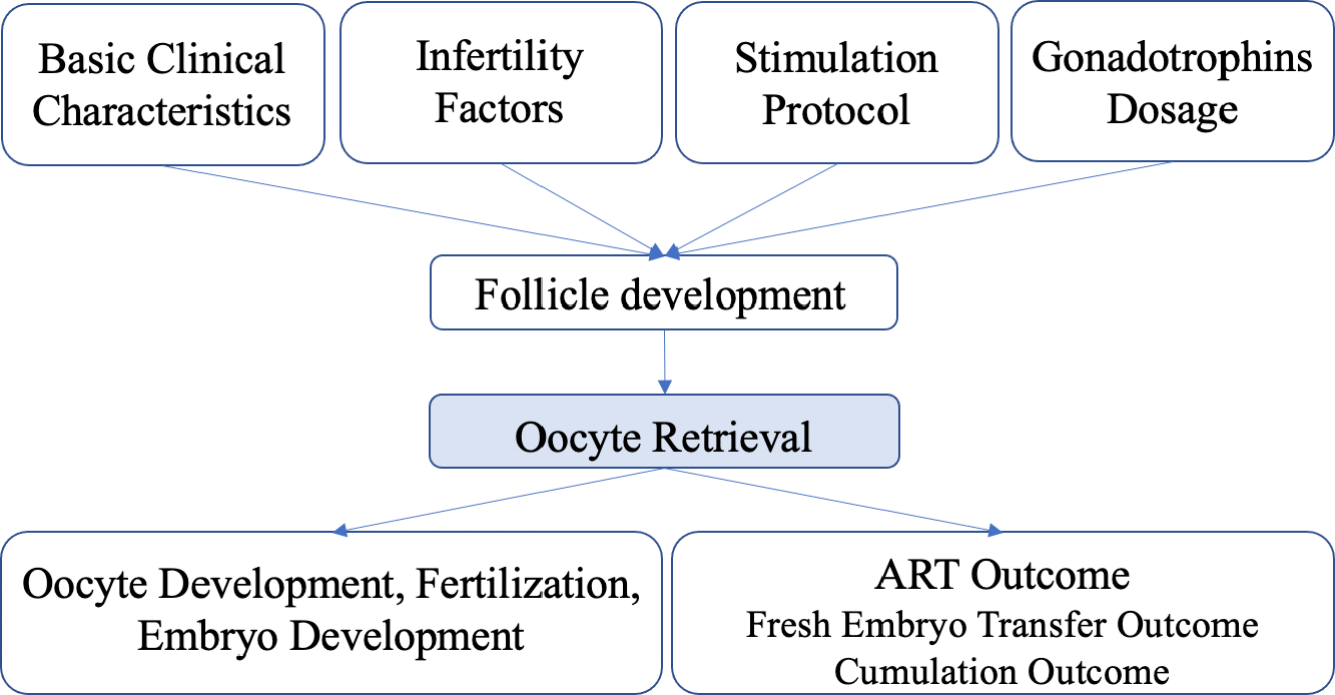
Flowchart of the study design.

Aspiration was performed first so that the follicle completely collapsed. Then flushing was performed to expand the follicle back to its original diameter in each round with a steady pressure of 125–140 mmHg. The flushing system was prefilled and prewarmed, and a mechanical pump with a foot pedal was used. Aspirated and flushed follicular fluid was collected into stacked tubes on a warming rack. The decision as to whether to perform flushing or not was made by the operator based on the following criteria: if the oocyte was obtained from the original follicular fluid, the follicular cavity would not be flushed; if the oocyte could not be acquired from the original follicular fluid, the follicular cavity would be flushed two to four times. The maximum number of flushes was six. The patients were divided into two groups based on whether the number of >14-mm follicles was consistent with the number of oocytes obtained: an oocyte retrieval satisfactory (ORS) group and an oocyte retrieval not satisfactory (ORNS) group. The causes of unsatisfactory oocyte retrieval and subsequent outcomes associated with it were analyzed (**Figure 1**).

### Data Collection

Basic clinical characteristics analyzed included age, body mass index, infertility duration, baseline follicle-stimulating hormone (FSH), baseline luteinizing hormone, baseline estradiol, and baseline progesterone. Infertility factors and the stimulation protocol used were also analyzed. Infertility factors were divided into six categories: multivariate fallopian tube factors; unexplained infertility (routine IVF examinations did not find any potential causes of infertility); other (uncertain single factors such as scarred uterus, benign ovarian cysts, simple oligomenorrhea, unicornuate uterus, mediastinal uterus, and uterine fibroids); male factors; artificial insemination by husband (AIH) or artificial insemination with donor semen (AID) failed; and polycystic ovary syndrome (PCOS). Because endometriosis and pelvic surgery can cause unsatisfactory oocyte retrieval, they were analyzed separately (13). The stimulation protocols included the follicular phase long-acting GnRH agonist long protocol, luteal phase short-acting GnRH agonist long protocol, antagonist protocol, mid-luteal phase long-acting GnRH agonist long protocol, early follicular phase long-acting GnRH agonist long protocol, and the short-term failed add long-acting GnRH agonist protocol.

On hCG day, the following were analyzed: total amount of FSH; total days of FSH; total amount of human menopausal gonadotropin (HMG); total days of HMG; levels of FSH, estradiol, and progesterone; number of >14-mm follicles under ultrasound; and average estradiol level of >14-mm follicles. hCG values after hCG was triggered were also analyzed. The number of oocytes retrieved was recorded, and then they were fertilized using the scheduled plan. Rescue ICSI was performed if the conventional fertilization method failed. The fertilization method, metaphase II rate, fertilization rate, cleavage rate, and high-quality embryo rate were recorded. Information pertaining to embryo transfer, the freezing of embryos, or a lack of transplantable embryos was also recorded.

Biochemical pregnancy tests were considered positive if the plasma concentration of β-hCG was ≥50 IU/L 14 to 18 days after embryo transplantation. Clinical pregnancy tests were considered positive if a fetal heartbeat was detected *via* ultrasound on day 35 after transplantation. At this time, if the gestational sac appeared in the uterus, it was considered an intrauterine pregnancy. The clinical outcomes recorded included clinical pregnancy rate for fresh embryo transfer, cumulative pregnancy rate (followed up to at least one clinical pregnancy or until all embryos were used; the percentage of cycles that resulted in at least one clinical pregnancy), and cumulative live birth rate (followed up to at least one live birth or until all embryos were used; the percentage of cycles that resulted in at least one live birth). Unpregnant cycles for which embryos were not used up until February 2019 were excluded when calculating the cumulative pregnancy and cumulative live birth rates. Pregnancy loss before 28 weeks of gestation was recorded as miscarriage. Cumulative pregnancy/live birth rate was the number of pregnancies/live births that had been achieved from the first to the current cycle divided by the initial number of patients, assuming no patient dropout (14).

### Statistical Analysis

Numerical variables are presented as means ± standard deviations, and categorical variables are presented as frequencies. Differences between two groups were compared *via t*-tests, and differences between three or more groups were compared *via* one-way analysis of variance. Differences between categorical variables were compared *via* chi-squared tests. All analyses were performed using SPSS 22.0 software (IBM Corp., Armonk, NY, USA). Differences were considered significant at *p <*0.05.

## Results

### Grouping Method and Baseline Characteristics

**Figure 2:**
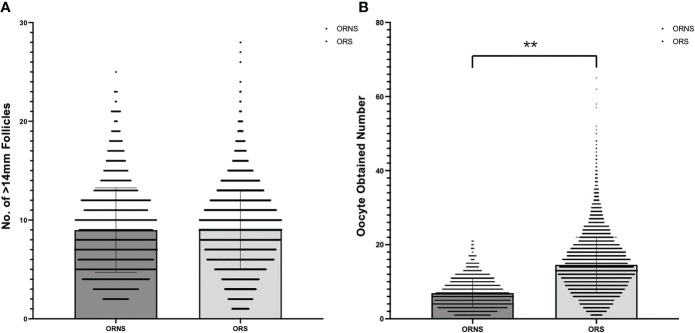
Frequency of >14-mm follicle number **(A)** and oocyte retrieval number **(B)** differences between the two groups (**P < 0.01).

**Figure 3:**
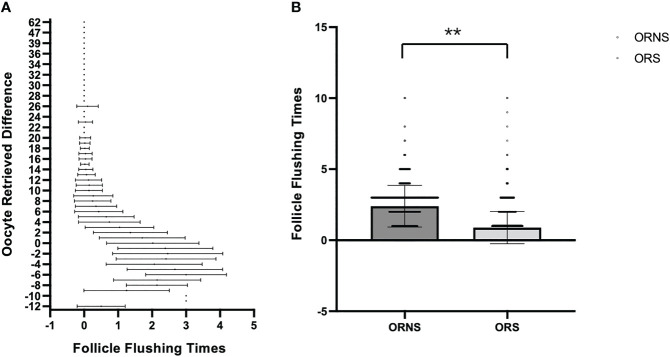
Relationship between follicular flushing and difference in oocytes obtained. **(A)** Relationship between follicular flushing times and difference in oocytes obtained. **(B)** Follicular flushing difference between the ORNS and ORS groups (**P < 0.01).

A total of 10,624 cycles were included in the study. Their ages ranged from 22 to 45 years. The oocyte numbers obtained ranged from 1 to 65. The oocyte numbers obtained and the frequencies of differences in oocyte numbers obtained in two groups are shown in **Figure 2**. During clinical procedures, if the number of oocytes obtained did not match, the operator usually subjectively increased follicular flushing times. The relationships between follicular flushing and different oocyte numbers obtained are shown in **Figure 3A** (*r* = 0.53, *p* < 0.01), and the follicular flushing times between the two groups are shown in **Figure 3B**.

In the ORS group, 9,330 oocytes were retrieved, and in the ORNS group, 1,294 oocytes were retrieved. The numbers of follicles >14 mm on hCG day were similar in the two groups. The significant indicators of reduced ovarian function that were higher in the ORNS group were age (32.78 ± 5.69 vs. 31.22 ± 5.33, *p* < 0.01), infertility duration (4.37 ± 3.58 vs. 3.86 ± 3.27, *p* < 0.01), baseline FSH (7.55 ± 3.31 vs. 6.77 ± 2.45, *p* < 0.01), and baseline estradiol (41.40 ± 25.92 vs. 39.58 ± 22.59, *p* < 0.01). Baseline progesterone was significantly lower in the ORNS group (0.59 ± 0.31 vs. 0.61 ± 0.30, *p* = 0.02) (**Table 1**, top). Of the six above-described infertility factors analyzed, only unexplained infertility was significantly lower in the ORNS group (11.36% vs. 14.34%, *p* < 0.01) (**Table 1**, middle). In comparison of endometriosis and pelvic surgery history, there was a significantly larger number of endometriosis patients in the ORNS group (5.26% vs. 3.59%, *p* < 0.01) (**Table 1**, bottom).

**Table 1:** Demographic and clinical characteristics of the study population.

Basal characteristics	Total	ORNS	ORS	*p*
No. of cycles	10,624	1,294	9,330	
No. of >14-mm follicles on hCG day	9.04 ± 4.04	8.98 ± 4.28	9.05 ± 4.01	0.61
Age (years)	31.41 ± 5.40	32.78 ± 5.69	31.22 ± 5.33	<0.01
BMI (kg/m^2^)	22.84 ± 3.15	22.76 ± 3.26	22.85 ± 3.14	0.38
Infertility duration (years)	3.92 ± 3.3	4.37 ± 3.58	3.86 ± 3.27	<0.01
Base FSH (mIU/ml)	6.87 ± 2.59	7.55 ± 3.31	6.77 ± 2.45	<0.01
Base LH (mIU/ml)	5.47 ± 3.42	5.34 ± 3.30	5.49 ± 3.44	0.14
Base E2 (pg/ml)	39.80 ± 23.02	41.40 ± 25.92	39.58 ± 22.59	<0.01
Base P (ng/ml)	0.61 ± 0.29	0.59 ± 0.31	0.61 ± 0.30	0.02
**Six main infertility factors**				
Multivariate	3,326	439 (33.93%)	2,887 (30.94%)	0.12
Fallopian tube factors	2,675	322 (24.88%)	2,353 (25.22%)	0.81
Unexplained infertility	1,485	147 (11.36%)	1,338 (14.34%)	0.01
Other factors	1,030	143 (11.05%)	887 (9.51%)	0.12
Male factors	1,130	129 (9.97%)	1,001 (10.73%)	0.48
AIH or AID failed	492	57 (4.40%)	435 (4.66%)	0.74
Other factors that may cause unsatisfactory oocyte retrieval				
PCOS	483	53 (4.10%)	430 (4.61%)	0.47
Endometriosis	403	68 (5.26%)	335 (3.59%)	<0.01
History of gynecology pelvic operation	1,718	201 (15.53%)	1,517 (16.26%)	0.60

Continuous data are presented as mean ± standard deviation for normally distributed data, or median and interquartile range for non-normally distributed data. Categorical data presented as number and percentage.

hCG, human chorionic gonadotropin; ORNS, oocyte retrieval not satisfactory; ORS, oocyte retrieval satisfactory.

### Stimulation Protocol Comparisons

In comparison of the six above-described stimulation protocols used in the two groups, the luteal phase short-acting GnRH agonist long protocol was used significantly more often in the ORNS group (28.59% vs. 15.62%, *p* < 0.01). Conversely, the follicular phase long-acting GnRH agonist long protocol was used significantly less often in the ORNS group (64.61% vs. 79.89%, *p* < 0.01). There were no significant differences in the rates of use of other stimulation protocols between the two groups (**Table 2**). Subgroup analysis of poor and normal response analysis is listed in **Supplementary Table 1**, and subgroup analyses of the three main stimulation protocols are listed in **Supplementary Table 2**.

**Table 2:** Stimulation protocol comparisons between the ORNS and ORS.

	Total	ORNS	ORS	*p*
Follicular phase long-acting GnRH agonist long protocol^a^	8,290	836 (64.61%)	7,454 (79.89%)	<0.01
Luteal phase short-acting GnRH agonist long protocol^b^	1,827	370 (28.59%)	1,457 (15.62%)	<0.01
Antagonist protocol^c^	307	38 (2.94%)	269 (2.88%)	0.98
Mid-luteal phase long-acting GnRH agonist long protocol^d^	98	34 (2.63%)	64 (0.69%)	<0.01
Early follicular phase long-acting GnRH agonist long protocol^e^	87	12 (0.93%)	75 (0.80%)	0.77
Short-term failed add long-acting GnRH agonist protocol^f^	15	4 (0.31%)	11 (0.12%)	0.18

In all protocols, Gn dosage was adjusted according to the growth of follicles until the trigger day.

a Follicular phase long-acting GnRH agonist long protocol: long-acting GnRH-a (3.75 mg) injected on the second day of menstruation for downregulation, and gonadotropins (Gn) were applied appropriately after 28–30 days.

b Luteal phase short-acting GnRH agonist long protocol: short-acting GnRH-a (0.1 mg) injected at mid-luteal phase or 16 days after combined oral contraceptives (COC) intake, and Gn were applied appropriately after 14 days.

c Antagonist protocol: applied Gn on the second day of menstruation and added GnRH-ant on the sixth day of menstruation until the trigger day.

d Mid-luteal phase long-acting GnRH agonist long protocol: first half dosage of long-acting GnRH-a (1.875 mg) injected at mid-luteal phase or 16 days after COC intake, second half dosage of long-acting GnRH-a (1.875 mg) injected 28 days after the first one, and Gn was applied appropriately after 14 days of the second half long-acting GnRH-a injection.

e Early follicular phase long-acting GnRH agonist long protocol: first long-acting GnRH-a injected on the second day of menstruation for downregulation, second long-acting GnRH-a injected 28 days after the first one, and Gn was applied appropriately 14 days after the second long-acting GnRH-a injection.

f Short-term failed add long-acting GnRH agonist protocol: a supplementary protocol for “luteal phase short-acting GnRH agonist long protocol”; if the downregulation was not effective (FSH > 5 mIU/ml or LH > 5 mIU/ml or a flare-up reaction was observed), long-acting GnRH-a (3.75 mg) was added and Gn was applied after 28–30 days.

### Gonadotropin Dosage and Hormone Level Comparisons

The total amount of FSH was significantly higher in the ORNS group than in the ORS group (2,071.03 ± 850.57 vs. 1,945.03 ± 674.50 IU, *p* < 0.01). The total number of days of FSH was significantly lower in ORNS group than in the ORS group (11.64 ± 3.05 vs. 12.39 ± 2.62, *p* < 0.01). The total amount of HMG was greater in the ORNS group (779.36 ± 921.04 vs. 628.38 ± 703.21 IU, *p* < 0.01), as was the total number of HMG days (5.02 ± 3.62 vs. 4.73 ± 2.98, *p* < 0.01). One day after hCG was triggered, luteinizing hormone was significantly higher in the ORNS group (1.29 ± 1.36 vs. 1.16 ± 1.45 U/L, *p* < 0.01). Estradiol was significantly lower (3,144.43 ± 1,986.05 vs. 3,642.19 ± 2,248.65, *p* < 0.01), as were the mean estradiol levels of >14-mm follicles (363.87 ± 167.13 vs. 418.42 ± 194.23, *p* < 0.01) and progesterone (0.84 ± 0.53 vs. 0.98 ± 1.17, *p* < 0.01) and the hCG value (134.250 ± 66.79 vs. 129.56 ± 63.03, *p* < 0.01) (**Table 3**).

**Table 3:** Gonadotropin dosage and duration comparisons between the ORNS group and the ORS group.

	Total	ORNS	ORS	*p*
Total amount of FSH* (IU)	1,960.38 ± 499.49	2,071.03 ± 850.57	1,945.03 ± 674.501	<0.01
Total days of FSH* (IU)	12.30 ± 2.69	11.64 ± 3.05	12.39 ± 2.62	<0.01
Total amount of HMG** (IU)	646.76 ± 734.82	779.36 ± 921.04	628.38 ± 703.21	<0.01
Total days of HMG**	4.77 ± 3.07	5.02 ± 3.62	4.73 ± 2.98	<0.01
LH on hCG day (mIU/ml)	1.18 ± 1.45	1.29 ± 1.36	1.16 ± 1.45	<0.01
E2 on hCG day (pg/ml)	3,581.56 ± 2,224.21	3,144.43 ± 1,986.05	3,642.19 ± 2,248.65	<0.01
P on hCG day (ng/ml)	0.96 ± 1.12	0.84 ± 0.53	0.98 ± 1.17	<0.01
Average E2 level of >14-mm follicles	411.78 ± 191.96	363.87 ± 167.13	418.42 ± 194.23	<0.01
hCG value (1 day after hCG triggered)	130.14 ± 63.52	134.250 ± 66.79	129.56 ± 63.03	<0.01

*Recombinant Human Follitropin for Injection (FSH), **Human Menopausal Gonadotropin for injection (HMG).

### Comparisons After Oocyte Retrieval to ART Outcome

The numbers of >14-mm follicles were similar in the ORNS and ORS groups after oocyte retrieval, but the follicular flushing times and numbers of oocytes obtained differed significantly [mean follicular flushing times 2.39 ± 1.47 (ORNS) vs. 0.88 ± 1.14 (ORS), *p* < 0.01; mean numbers of oocytes retrieved 6.93 ± 3.87 vs. 14.57 ± 7.46, *p* < 0.01). The rates of IVF (71.41% vs. 71.60%), ICSI (26.43% vs. 25.56%), and rescue ICSI (2.16% vs. 2.84%) were similar in the two groups. The cleavage rate was similar in the two groups (98.85% ± 6.51% vs. 98.72% ± 4.70%), but the metaphase II rate, fertilization rate, and high-quality embryo rate were all significantly higher in the ORNS group than in the ORS group (metaphase II rate 85.00% ± 17.15% vs. 81.00% ± 15.52%, *p* < 0.01; fertilization rate 72.88% ± 22.22% vs. 67.73% ± 19.73%, *p* < 0.01; high-quality embryo rate 68.78% ± 28.43% vs. 56.05% ± 25.98%, *p* < 0.01). In the ORNS group, there were more fresh embryo transfer cycles (86.48% vs. 76.05%, *p* < 0.01), and the rate of no transferable embryos was higher (0.70% vs. 0.33%, *p* < 0.01), whereas in the ORS group, there were more freeze-all cycles (11.59% vs. 23.58%, *p* < 0.01). Endometrial thickness on embryo transfer (ET) day was lower in the ORNS group than in the ORS group (11.66 ± 2.51 vs. 11.93 ± 2.59, *p* < 0.01). Fresh cycles, biochemical pregnancy rate, clinical pregnancy rate, intrauterine pregnancy rate, miscarriage rate, and live birth rate were all similar in the two groups. The cumulative pregnancy rate and cumulative live birth rate were significantly lower in the ORNS group (**Table 4**).

**Table 4:** Comparisons between the ORNS and ORS after oocyte retrieval to embryo transfer.

	Total	ORNS	ORS	*p*
Follicular flushing times	1.07 ± 1.28	2.39 ± 1.47	0.88 ± 1.14	<0.01
No. of retrieved oocytes	13.64 ± 7.55	6.93 ± 3.87	14.57 ± 7.46	<0.01
Fertilization method: IVF	7,604	924 (71.41%)	6,680 (71.60%)	0.97
Fertilization method: ICSI	2,727	342 (26.43%)	2,385 (25.56%)	0.63
Fertilization method: rescue ICSI	293	28 (2.16%)	265 (2.84%)	0.20
MII rate (%)	81.48% ± 1.75%	85.00% ± 17.15%	81.00% ± 15.52%	<0.01
Fertilization rate (%)	68.36% ± 20.12%	72.88% ± 22.22%	67.73% ± 19.73%	<0.01
Cleavage rate (%)	98.73% ± 4.96%	98.85% ± 06.51%	98.72% ± 04.70%	0.41
High-quality embryonic rate (%)	57.60% ± 26.62%	68.78% ± 28.43%	56.05% ± 25.98%	<0.01
Fresh embryo transfer cycle	8,214	1,119 (86.48%)	7,095 (76.05%)	<0.01
Freeze-all cycle	2,350	150 (11.59%)	2,200 (23.58%)	<0.01
No transferable cycle	40	9 (0.70%)	31 (0.33%)	<0.01
Endometrial thickness on ET day	11.9 ± 2.58	11.66 ± 2.51	11.93 ± 2.59	<0.01
Biochemical pregnancy	5,190	609 (47.06%)	4,581 (49.10%)	0.43
Clinical pregnancy rate for fresh embryo transfer (%)	4,790	561 (43.35%)	4,229 (45.33%)	0.42
Intrauterine pregnancy	4,665	549 (42.43%)	4,116 (44.12%)	0.48
Miscarriage rate	640	80 (6.18%)	560 (6.00%)	0.85
Live birth rate	3,933	456 (35.24%)	3,447 (36.95%)	0.43
Cumulative pregnancy rate (%)	7,604	741 (57.26%)	6,863 (73.56%)	<0.01
Cumulative live birth rate (%)	6,751	642 (49.61%)	6,109 (65.48%)	<0.01

## Discussion

Oocyte retrieval is a routine procedure during ART, but technical difficulties associated with oocyte retrieval and subsequent unsatisfactory numbers of oocytes obtained have rarely been reported. Follicles >14 mm as determined *via* ultrasound are usually considered to contain a mature oocyte. In the current study, however, in 12.18% of cycles (1,294/10,624), the final number of oocytes obtained was less than the number of >14-mm follicles on hCG day. Thus, all cycles were divided into two groups (ORNS vs. ORS) based on this standard. Data showed ORNS patients tended to be older, have a longer duration of infertility, and have higher FSH, indicating worse ovarian reserve and a higher incidence of infertility caused by endometriosis. There were also significant differences in stimulation protocols between the two groups. The use of a follicular phase long-acting GnRH agonist long protocol was more common in the ORS group. It is reasonable that the ORNS group used more gonadotropins due to a poor ovarian reserve, but the ORNS group had fewer total FSH days than the ORS group. On hCG day, the ORNS group had higher luteinizing hormone and lower estradiol and progesterone. After oocyte retrieval, even though the obtained oocyte numbers were significantly lower in the ORNS group, the proportion of high-quality embryos was even higher than ORS cycles. An unsatisfactory oocyte retrieval number did not influence the biochemical pregnancy rate, clinical pregnancy rate, intrauterine pregnancy rate, miscarriage rate, or live birth rate during fresh cycles, but it was associated with the cumulative pregnancy rate and the cumulative live birth rate.

Ovarian dysfunction is one of the biggest contributors to an unsatisfactory number of oocytes being retrieved and a poor ART outcome. In the present study, of the 1,040 poor response cycles, 38.17% (397/1,040) were ORNS cycles, while in the normal response cycles, this was only 9.36% (897/9,584). This also indicated that ovarian dysfunction patients were more likely to experience ORNS. Besides, poor responders cannot be regarded as belonging to the same populations (15). A systematic review of 19 poor ovarian responder studies indicated that even though the final consequence was lower pregnancy, and further pregnancy prospects were reduced when fewer oocytes were retrieved, if >2 oocytes were retrieved, the poor responders exhibited a more favorable outcome (15). That study further illustrated that an insufficient number of oocytes obtained is associated with pregnancy rate, especially in poor ovarian responders. There are usually a greater focus on follicle development during ovarian stimulation and a little focus on the how many follicles contain mature oocytes and can be considered as “an actual follicle.” After ovarian stimulation, the ORNS group had lower average estradiol levels and higher hCG levels, indicating that during follicle development the function of granulosa cells was not as good as that in the ORS group. It may also be that those small follicles contributed to the estradiol level, especially in the ORS group. The specific reason remains to be further investigated. The surgical records of these patients also provided some potentially relevant information. They usually described poor follicle surface tension, not enough granulosa cells observed, variable flushing times without oocytes harvested, premature rupture of some follicles, and sticky follicular fluid. Thus, the results of the current study provide evidence for future research on follicle development and infertility treatment.

Ovarian stimulation is an extremely important component of IVF. It is closely related to the quality of oocytes and embryos and the occurrence of IVF complications. Two main hyperstimulation protocols are currently used: the follicular phase long-acting GnRH agonist long protocol and the luteal phase short-acting GnRH agonist long protocol. The latter is considered a classical ovarian stimulation protocol due to its stable successful rate. Notably, however, in-depth research of downregulation indicates that long-acting GnRH agonist protocol can improve the receptivity of the endometrium and increase the embryo implantation rate, significantly improving the clinical pregnancy rate associated with IVF cycles (16). Therefore, the long-acting GnRH protocol and long-term follicular phase have been widely used. In the present study, it was associated with an additional advantage: a greater number of mature follicles obtained.

Endometriosis is a relatively clear cause of oocyte retrieval difficulty (11), which may be related to insufficient cumulus expansion (12). Endometriosis may also interfere with the ovarian response during hyper-stimulation by follicular fluid contaminated with endometrioma content (17). In a recent meta-analysis, lower numbers of mature oocytes were retrieved in patients with endometriosis (18). Our conclusions were consistent with these studies, further illustrating the negative effects of endometriosis on ART.

However, no article clearly pointed out which follicle size had matured oocytes, or which follicle size had unmatured oocytes, indicating no aspiration value. Several studies provided the relationship between follicle size and oocyte development potential (19–22). From these studies, we found that the size of the follicle and the maturation of oocytes were relative. Larger follicles had a higher chance of having mature oocytes. In our center, all >12-mm follicles would be recorded and aspirated. However, >14-mm follicle numbers would be used to evaluate whether the retrieval process was satisfactory (the actual obtained oocyte number should be same or >14-mm follicle number). If the obtained oocyte number reached or exceeded the >14-mm follicle number, the operator could finish aspiration. Otherwise, the operator would increase the flushing times or puncture smaller follicles. Follicular flushing is widely used in ART centers. Over one-third of the centers (38%, 9/24) performed selective follicular flushing in poor responders or those with other specific indications (e.g., when follicular aspiration did not yield oocytes at the beginning of the operation). Two centers (8%) did not perform follicular flushing (2). Randomized controlled trial results have demonstrated the safety of follicular flushing in normal and poor responders (3–9), and in observational studies, follicular flushing increased oocyte yield, which was associated with a higher cumulative live birth rate (23). Meta-analyses have concluded that flushing did not change the oocyte yield (24–26). At our clinic, follicular flushing was used in 53.2% of the cycles. It may be useful for ART centers to investigate the value of this practice. All these data indicate that follicular flushing is safe and that it does not affect the rate of retrieval of a satisfactory number of oocytes.

In conclusion, ovarian dysfunction patients were more likely to experience ORNS. The follicular phase long-acting GnRH agonist long protocol was more advanced to reduce the occurrence of oocyte retrieval difficulty. Although ORNS does not affect embryo quality or the fresh cycle pregnancy rate, it significantly reduces the cumulative pregnancy rate due to fewer transplantable embryos.

## Data Availability Statement

The original contributions presented in the study are included in the article/**Supplementary Material**. Further inquiries can be directed to the corresponding author.

## Ethics Statement

The studies involving human participants were reviewed and approved by the Independent Ethics Committee of Zhengzhou University. The patients/participants provided their written informed consent to participate in this study.

## Author Contributions

YW and MZ participated in designing the study. SY and QL recruited the subjects and collected the data. HS analyzed the data. YW and MZ wrote the manuscript. All authors have reviewed the data and analysis and contributed to the revision of the manuscript. The final manuscript and the order of authorship have been approved by all authors.

## Funding

This work was supported by National Natural Science Foundation of China (No.82101742) for MZ , Henan Province Medical Science and Technology Co-construction Project for HS and MZ, the Youth Innovation Fund of the First Affiliated Hospital of Zhengzhou University for YW. This work was also supported by the National Natural Science Foundation of China for the National Key R&D Program of China (2019YFA 0110900) and the International (Regional) Cooperation and Exchange Projects (81820108016) to Y-pS.

## Conflict of Interest

The authors declare that the research was conducted in the absence of any commercial or financial relationships that could be construed as a potential conflict of interest.

## Publisher’s Note

All claims expressed in this article are solely those of the authors and do not necessarily represent those of their affiliated organizations, or those of the publisher, the editors and the reviewers. Any product that may be evaluated in this article, or claim that may be made by its manufacturer, is not guaranteed or endorsed by the publisher.

## Supplementary Material

Supplementary Table 1 Subgroup analysis of poor and normal respond cycles in ORNS and ORS. *Chi squre compare the ratio of ORNS and ORS in poor responder and normal responder groups.

Supplementary Table 2 Subgroup analysis of different stimulation protocols ORNS and ORS. *Chi squre compare the ratio of ORNS and ORS in poor responder and normal responder groups.
