# Supplementary material for: Causes and Effects of Oocyte Retrieval Difficulties: A Retrospective Study of 10,624 Cycles
Source: Front Endocrinol (Lausanne). 2022 Jan 3;12:564344. doi: 10.3389/fendo.2021.564344 (PMC8761769; doi:10.3389/fendo.2021.564344)
Supplement: Supplementary Table 1 — Subgroup analysis of poor and normal respond cycles in ORNS and ORS. *Chi squre compare the ratio of ORNS and ORS in poor responder and normal responder groups. [file Table_1.docx]

**Supplementary Table 1 Subgroup analysis of poor and normal respond cycles in ORNS and ORS**

| **Poor Responders** | **Total** | **ORNS** | **ORS** | ***P*** |
| --- | --- | --- | --- | --- |
| No. of cycles | 1040 | 397(38.17%) | 643(61.83%) | <0.01* |
| No. of >14mm follicles on HCG day | 3.15±1.74 | 4.61±1.7 | 2.25±0.99 | <0.01 |
| Age (years) | 36.62±5.49 | 35.69±5.51 | 37.19±5.4 | <0.01 |
| BMI(kg/m^2^) | 23.05±2.98 | 22.94±3.11 | 23.11±2.9 | 0.36 |
| Infertility duration (years) | 4.79±4.22 | 4.62±3.86 | 4.9±4.43 | 0.29 |
| Base FSH(mIU/ml) | 9.42±4.55 | 9.07±3.98 | 9.64±4.86 | 0.05 |
| Base E2(pg/ml) | 45.25±30.28 | 43.07±30.21 | 46.59±30.27 | 0.07 |
| Base P(ng/ml) | 0.56±0.31 | 0.55±0.31 | 0.56±0.3 | 0.81 |
| Base LH(mIU/ml) | 4.74±2.6 | 4.73±2.45 | 4.74±2.69 | 0.98 |
| Total amount of FSH | 2270.7±906.19 | 2396.09±922.33 | 2193.28±888 | <0.01 |
| Total days of FSH | 9.86±3.56 | 10.7±3.49 | 9.35±3.51 | <0.01 |
| Total amount of HMG | 1214.68±1096.24 | 1191.5±1138.38 | 1228.99±1070.04 | 0.59 |
| Total days of HMG | 6.24±3.95 | 6.38±4.19 | 6.15±3.8 | 0.37 |
| LH on HCG day(mIU/ml) | 2.37±2.71 | 1.7±1.57 | 2.78±3.15 | <0.01 |
| E2 on HCG day(pg/ml) | 1318.69±811.96 | 1808.65±949.39 | 1016.18±520.46 | <0.01 |
| P on HCG day(ng/ml) | 0.54±0.39 | 0.65±0.41 | 0.47±0.36 | <0.01 |
| Average E2 level of >14mm follicles (pg/ml) | 454.83±214.41 | 405.85±193.14 | 485.07±221.34 | <0.01 |
| Follicular flushing times | 3.04±1.33 | 3.49±1.35 | 2.76±1.24 | <0.01 |
| No. of retrieved oocytes | 2.87±1.05 | 2.83±1.07 | 2.9±1.03 | 0.27 |
| MII rate (%) | 87.94%±19.17% | 89.29%±18.64% | 87.10%±19.46% | 0.07 |
| Fertilization rate (%) | 78.42%±23.81% | 79.70%±23.46% | 77.63%±24.00% | 0.17 |
| Cleavage rate (%) | 99.10%±6.76% | 98.91%±7.78% | 99.22%±6.05% | 0.47 |
| High-quality embryonic rate (%) | 78.09%±29.03% | 79.81%±27.72% | 77.02%±29.79% | 0.13 |
| Endometrial thickness on ET day | 10.94±2.68 | 11.22±2.62 | 10.77±2.71 | <0.01 |
| Biochemical pregnancy | 350 | 135 | 215 | 0.94 |
| Clinical pregnancy rate for fresh embryo transfer (%) | 299 | 115 | 184 | 0.98 |
| Intrauterine pregnancy | 291 | 112 | 179 | 0.98 |
| Miscarriage rate | 76 | 22 | 54 | 0.14 |
| Live birth rate | 206 | 85 | 121 | 0.45 |
| Cumulative pregnancy rate (%) | 346 | 127 | 219 | 0.67 |
| Cumulative live birth rate (%) | 236 | 96 | 140 | 0.52 |
|  |  |  |  |  |
| **Normal Responders** | **Total** | **ORNS** | **ORS** | ***P*** |
| No. of cycles | 9584 | 897(9.36%) | 8687(90.64%) | <0.01* |
| No. of >14mm follicles on HCG day | 9.68±3.69 | 10.92±3.59 | 9.55±3.67 | <0.01 |
| Age (years) | 30.85±5.08 | 31.49±5.28 | 30.78±5.05 | <0.01 |
| BMI(kg/m^2^) | 22.81±3.17 | 22.68±3.32 | 22.83±3.15 | <0.01 |
| Infertility duration (years) | 3.82±3.18 | 4.26±3.44 | 3.78±3.15 | <0.01 |
| Base FSH(mIU/ml) | 6.60±2.09 | 6.87±2.71 | 6.57±2.01 | <0.01 |
| Base E2(pg/ml) | 39.21±22.01 | 40.67±23.75 | 39.06±21.82 | 0.04 |
| Base P(ng/ml) | 0.62±0.3 | 0.6±0.3 | 0.62±0.3 | 0.09 |
| Base LH(mIU/ml) | 5.55±3.49 | 5.61±3.58 | 5.55±3.48 | 0.61 |
| Total amount of FSH | 1926.71±664.66 | 1927.16±774.93 | 1926.66±652.26 | 0.98 |
| Total days of FSH | 12.56±2.44 | 12.06±2.73 | 12.61±2.4 | <0.01 |
| Total amount of HMG | 585.14±655.34 | 596.95±736.8 | 583.92±646.38 | 0.57 |
| Total days of HMG | 4.61±2.91 | 4.41±3.16 | 4.63±2.89 | 0.04 |
| LH on HCG day(mIU/ml) | 1.05±1.16 | 1.11±1.22 | 1.04±1.16 | 0.10 |
| E2 on HCG day(pg/ml) | 3827.11±2190.09 | 3735.62±2037.95 | 3836.56±2205.1 | 0.19 |
| P on HCG day(ng/ml) | 1.01±1.16 | 0.93±0.55 | 1.02±1.2 | 0.03 |
| Average E2 level of >14mm follicles (pg/ml) | 407.1±188.79 | 345.3±150.66 | 413.49±191.16 | <0.01 |
| Follicular flushing times | 0.85±1.08 | 1.91±1.24 | 0.74±1 | <0.01 |
| No. of retrieved oocytes | 14.81±7 | 8.75±3.21 | 15.44±6.99 | <0.01 |
| MII rate (%) | 80.79%±15.20% | 83.11%±16.10% | 80.55%±15.09% | <0.01 |
| Fertilization rate (%) | 67.26%±19.36% | 69.86%±20.96% | 67.00%±19.17% | <0.01 |
| Cleavage rate (%) | 98.69%±4.72% | 98.82%±5.87% | 98.68%±4.59% | 0.40 |
| High-quality embryonic rate (%) | 55.38%±25.37% | 63.90%±27.37% | 54.50%±24.99% | <0.01 |
| Endometrial thickness on ET day | 12.00±2.55 | 11.85±2.44 | 12.02±2.06 | 0.69 |
| Biochemical pregnancy | 4840 | 474 | 4366 | 0.41 |
| Clinical pregnancy rate for fresh embryo transfer (%) | 4491 | 446 | 4045 | 0.30 |
| Intrauterine pregnancy | 4374 | 437 | 3937 | 0.25 |
| Miscarriage rate | 564 | 58 | 506 | 0.51 |
| Live birth rate | 3727 | 371 | 3356 | 0.31 |
| Cumulative pregnancy rate (%) | 7258 | 614 | 6644 | 0.05 |
| Cumulative live birth rate (%) | 6015 | 518 | 5497 | 0.12 |
| * Chi squre compare the ratio of ORNS and ORS in poor responder and normal responder groups | | |  |  |
