# Supplementary material for: Causes and Effects of Oocyte Retrieval Difficulties: A Retrospective Study of 10,624 Cycles
Source: Front Endocrinol (Lausanne). 2022 Jan 3;12:564344. doi: 10.3389/fendo.2021.564344 (PMC8761769; doi:10.3389/fendo.2021.564344)
Supplement: Supplementary Table 2 — Subgroup analysis of different stimulation protocols ORNS and ORS. *Chi squre compare the ratio of ORNS and ORS in poor responder and normal responder groups. [file Table_2.docx]

| **Supplementary Table 2 Subgroup analysis of different stimulation protocols ORNS and ORS** | | | | | |  | |
| --- | --- | --- | --- | --- | --- | --- | --- |
| **Follicular phase long-acting GnRH-agonist long protocol** | **Total** | **ORNS** | | **ORS** | | ***P*** | |
| No. of cycles | 8290 | 836 | | 7454 | |  | |
| No. of >14mm follicles on HCG day | 9.38±3.78 | 9.47±4.06 | | 9.37±3.75 | | *0.449* | |
| Age (years) | 30.93±5.10 | 32.39±5.43 | | 30.77±5.04 | | <0.01 | |
| BMI(kg/m^2^) | 22.86±3.18 | 22.74±3.27 | | 22.87±3.17 | | 0.29 | |
| Infertility duration (years) | 3.82±3.20 | 4.29±3.53 | | 3.77±3.15 | | <0.01 | |
| Base FSH(mIU/ml) | 6.62±2.18 | 7.23±3.11 | | 6.55±2.04 | | <0.01 | |
| Base E2(pg/ml) | 39.76±21.88 | 41.99±25.09 | | 39.52±21.48 | | <0.01 | |
| Base P(ng/ml) | 0.62±0.30 | 0.6±0.31 | | 0.62±0.29 | | 0.03 | |
| Base LH(mIU/ml) | 5.54±3.47 | 5.36±3.27 | | 5.56±3.49 | | 0.11 | |
| Total amount of FSH | 1961.35±604.32 | 2093.34±679.26 | | 1946.54±593.55 | | <0.01 | |
| Total days of FSH | 12.92±2.07 | 12.5±2.09 | | 12.96±2.07 | | <0.01 | |
| Total amount of HMG | 667.57±668.41 | 841.87±795.2 | | 648.02±649.81 | | <0.01 | |
| Total days of HMG | 5.02±2.86 | 5.44±3.23 | | 4.97±2.81 | | <0.01 | |
| LH on HCG day(mIU/ml) | 0.98±1.24 | 1.07±1.39 | | 0.97±1.23 | | 0.33 | |
| E2 on HCG day(pg/ml) | 3523.97±2061.15 | 2986.61±1754.87 | | 3584.22±2084.18 | | <0.01 | |
| P on HCG day(ng/ml) | 1.01±1.24 | 0.88±0.57 | | 1.03±1.29 | | <0.01 | |
| Average E2 level of >14mm follicles (pg/ml) | 389.35±183.98 | 327.51±148.21 | | 396.29±186.3 | | <0.01 | |
| Follicular flushing times | 0.89±1.78 | 2.25±1.43 | | 0.74±1.04 | | <0.01 | |
| No. of retrieved oocytes | 14.59±7.38 | 7.37±3.68 | | 15.4±7.25 | | <0.01 | |
| MII rate (%) | 81.39%±15.18% | 84.61%±16.73% | | 81.03%±14.95% | | <0.01 | |
| Fertilization rate (%) | 67.13%±19.56% | 70.46%±21.53% | | 66.75%±19.29% | | <0.01 | |
| Cleavage rate (%) | 98.77%±4.54% | 99.03%±5.20% | | 98.74%±4.46% | | 0.128 | |
| High-quality embryonic rate (%) | 56.79%±25.71% | 68.69%±27.09% | | 55.42%±25.20% | | <0.01 | |
| Endometrial thickness on ET day | 12.07±0.55 | 11.82±2.49 | | 12.10±2.55 | | 0.02 | |
| Biochemical pregnancy | 4322 | 447(53.47%) | | 3875(51.99%) | | 0.67 | |
| Clinical pregnancy rate for fresh embryo transfer (%) | 4019 | 418(50.00%) | | 3601(48.31%) | | 0.61 | |
| Intrauterine pregnancy | 3918 | 410(49.04%) | | 3508(47.06%) | | 0.54 | |
| Miscarriage rate | 497 | 54(6.46%) | | 443(5.94%) | | 0.63 | |
| Live birth rate | 3344 | 344(41.15%) | | 3000(40.25%) | | 0.79 | |
| Cumulative pregnancy rate (%) | 6286 | 519(62.08%) | | 5767(77.37%) | | <0.01 | |
| Cumulative live birth rate (%) | 5222 | 433(51.79%) | | 4789(64.25%) | | <0.01 | |
|  |  |  | |  | |  | |
| **Luteal phase short-acting GnRH-agonist long protocol** | **Total** | **ORNS** | | **ORS** | | ***P*** | |
| No. of cycles | 1827 | 370 | | 1457 | |  | |
| No. of >14mm follicles on HCG day | 8.75±4.49 | 8.68±4.48 | | 8.77±4.5 | | *0.742* | |
| Age (years) | 32.32±5.83 | 33.2±6.08 | | 32.1±5.75 | | <0.01 | |
| BMI(kg/m^2^) | 22.74±3.05 | 22.81±3.28 | | 22.72±2.99 | | 0.62 | |
| Infertility duration (years) | 4.20±3.54 | 4.62±3.74 | | 4.09±3.48 | | 0.01 | |
| Base FSH(mIU/ml) | 7.33±2.81 | 7.78±2.92 | | 7.22±2.77 | | <0.01 | |
| Base E2(pg/ml) | 39.59±25.85 | 41.73±27.2 | | 39.04±25.47 | | 0.07 | |
| Base P(ng/ml) | 0.58±0.30 | 0.57±0.3 | | 0.58±0.3 | | 0.08 | |
| Base LH(mIU/ml) | 5.33±3.34 | 5.41±3.49 | | 5.31±3.3 | | 0.61 | |
| Total amount of FSH | 2140.25±869.24 | 2270.34±943.98 | | 2107.22±846.38 | | <0.01 | |
| Total days of FSH | 11.14±1.94 | 11.28±2.01 | | 11.11±1.92 | | 0.125 | |
| Total amount of HMG | 272.11±475.88 | 301.52±579.77 | | 264.64±445.59 | | 0.254 | |
| Total days of HMG | 2.81±2.39 | 2.97±2.73 | | 2.77±2.3 | | 0.197 | |
| LH on HCG day(mIU/ml) | 1.63±0.93 | 1.59±0.84 | | 1.65±0.95 | | 0.325 | |
| E2 on HCG day(pg/ml) | 4284.12±2724.35 | 3703.61±2368.86 | | 4431.54±2789.02 | | <0.01 | |
| P on HCG day(ng/ml) | 0.82±0.45 | 0.79±0.46 | | 0.83±0.45 | | 0.11 | |
| Average E2 level of >14mm follicles (pg/ml) | 496.45±129.49 | 433.83±184.44 | | 512.35±191.31 | | <0.01 | |
| Follicular flushing times | 1.51±1.36 | 2.54±1.4 | | 1.25±1.22 | | <0.01 | |
| No. of retrieved oocytes | 11.41±7.16 | 6.66±4.08 | | 12.61±7.28 | | <0.01 | |
| MII rate (%) | 10.91%±17.43% | 84.86%±17.97% | | 79.91%±17.16% | | <0.01 | |
| Fertilization rate (%) | 71.49%±21.00 | 75.85%±22.64% | | 70.39%±20.42% | | <0.01 | |
| Cleavage rate (%) | 98.44%±6.51% | 98.29%±9.14% | | 98.48%±5.66% | | 0.61 | |
| High-quality embryonic rate (%) | 57.68%±28.57% | 66.07%±30.55% | | 55.55%±27.65% | | <0.01 | |
| Endometrial thickness on ET day | 11.46±2.51 | 11.39±2.46 | | 11.47±2.53 | | 0.6 | |
| Biochemical pregnancy | 708 | 130(35.14%) | | 578(39.67%) | | 0.31 | |
| Clinical pregnancy rate for fresh embryo transfer (%) | 625 | 113(30.54%) | | 512(35.14%) | | 0.26 | |
| Intrauterine pregnancy | 607 | 110(29.73%) | | 497(34.11%) | | 0.27 | |
| Miscarriage rate | 105 | 17(4.59%) | | 88(6.04%) | | 0.37 | |
| Live birth rate | 489 | 92(24.86%) | | 397(27.25%) | | 0.52 | |
| Cumulative pregnancy rate (%) | 1106 | 184(49.73%) | | 922(63.28%) | | 0.02 | |
| Cumulative live birth rate (%) | 881 | 153(41.35%) | | 728(49.97%) | | 0.08 | |
|  |  |  | |  | |  | |
| **Antagonist protocol** | **Total** | **ORNS** | | **ORS** | | ***P*** | |
| No. of cycles | 307 | 38 | | 269 | |  | |
| No. of >14mm follicles on HCG day | 2.53±1.58 | 3.11±1.43 | | 2.45±1.58 | | *0.02* | |
| Age (years) | 38.61±5.17 | 37.74±5.7 | | 38.73±5.09 | | 0.27 | |
| BMI(kg/m^2^) | 22.96±2.73 | 23.13±2.75 | | 22.94±2.73 | | 0.69 | |
| Infertility duration (years) | 4.86±4.43 | 4.29±4 | | 4.94±4.48 | | 0.40 | |
| Base FSH(mIU/ml) | 10.74±5.68 | 12.04±6.35 | | 10.56±5.56 | | 0.13 | |
| Base E2(pg/ml) | 47.35±32.31 | 40.36±31.28 | | 48.33±32.39 | | 0.15 | |
| Base P(ng/ml) | 0.56±0.32 | 0.54±0.35 | | 0.57±0.31 | | 0.60 | |
| Base LH(mIU/ml) | 4.84±2.77 | 4.94±2.61 | | 4.83±2.79 | | 0.81 | |
| Total amount of FSH | 1612.38±921.48 | 1642.11±1098.37 | | 1608.18±896.01 | | 0.83 | |
| Total days of FSH | 7.15±3.73 | 7.21±4.49 | | 7.14±3.61 | | 0.91 | |
| Total amount of HMG | 1260.95±1019.87 | 1474.34±1078.61 | | 1230.81±1009.77 | | 0.17 | |
| Total days of HMG | 6.03±3.67 | 6.87±4.03 | | 5.91±3.61 | | 0.13 | |
| LH on HCG day(mIU/ml) | 4.16±3.67 | 3.72±2.45 | | 4.22±3.81 | | 0.44 | |
| E2 on HCG day(pg/ml) | 1158.23±753.71 | 1241.94±729.73 | | 1146.4±757.61 | | 0.47 | |
| P on HCG day(ng/ml) | 0.63±0.38 | 0.64±0.33 | | 0.62±0.39 | | 0.77 | |
| Average E2 level of >14mm follicles (pg/ml) | 498.38±224.37 | 403.17±150.25 | | 511.83±229.99 | | 0.01 | |
| Follicular flushing times | 2.80±1.47 | 3.97±1.97 | | 2.63±1.31 | | <0.01 | |
| No. of retrieved oocytes | 3.62±2.85 | 1.68±0.93 | | 3.89±2.92 | | <0.01 | |
| MII rate (%) | 88.28%±18.63% | 96.49%±12.35% | | 87.13%±19.09% | | <0.01 | |
| Fertilization rate (%) | 79.13%±23.39% | 93.86%±16.63% | | 77.05%±23.48% | | <0.01 | |
| Cleavage rate (%) | 99.18%±5.71% | 100.00%±0.00% | | 99.07%±6.09% | | 0.347 | |
| High-quality embryonic rate (%) | 78.33%±28.34% | 91.67%±24.43% | | 76.45%±28.96% | | <0.01 | |
| Endometrial thickness on ET day | 9.86±2.39 | 10.32±2.90 | | 9.79±2.31 | | 0.21 | |
| Biochemical pregnancy | 56 | 6(15.79%) | | 50(18.59%) | | 0.90 | |
| Clinical pregnancy rate for fresh embryo transfer (%) | 46 | 6(15.79%) | | 40(14.87%) | | 0.91 | |
| Intrauterine pregnancy | 43 | 5(13.16%) | | 38(14.13%) | | 0.91 | |
| Miscarriage rate | 16 | 2(5.26%) | | 14(5.20%) | | 0.71 | |
| Live birth rate | 26 | 3(7.89%) | | 23(8.55%) | | 0.85 | |
| Cumulative pregnancy rate (%) | 83 | 12(31.58%) | | 71(26.39%) | | 0.75 | |
| Cumulative live birth rate (%) | 46 | 7(18.42%) | | 39(14.50%) | | 0.06 | |
| * Chi squre compare the ratio of ORNS and ORS in poor responder and normal responder groups | | |  | |  | |  |
